# Temporal Trends of Age-Adjusted Mortality Rates for Rheumatic Heart Disease in Brazil From 2000 to 2021

**DOI:** 10.7759/cureus.52322

**Published:** 2024-01-15

**Authors:** Billy McBenedict, Zaeemah Mansoor, Abhishek Chaudhary, Anusha Thomas, Muhammad Yaseen, Wilhelmina Hauwanga

**Affiliations:** 1 Medicine, Hospital Universitário Antônio Pedro (Antonio Pedro University Hospital), Niteroi, BRA; 2 Faculty of Health Sciences, Karachi Medical and Dental College, Karachi, PAK; 3 Medicine, Smt. NHL Municipal Medical College, Ahmedabad, IND; 4 Neurology, Christian Medical College and Hospital, Ludhiana, IND; 5 Medicine and Surgery, Gambat Institute of Medical Sciences, Gambat, PAK; 6 Family Medicine, Faculty of Medicine, Federal University of the State of Rio de Janeiro, Rio de Janeiro, BRA

**Keywords:** time series analysis, joinpoint regression, acute rheumatic heart disease, chronic rheumatic heart disease, rheumatic fever, rheumatic heart disease

## Abstract

Background

Rheumatic heart disease (RHD) is a chronic cardiovascular condition stemming from an infectious origin, posing a substantial health burden, particularly in economically disadvantaged regions. It starts with acute rheumatic fever (ARF), a complication following group A Streptococcus infection, leading to heart valve damage and, over time, structural heart abnormalities. RHD contributes to premature deaths, especially in low-middle-income countries. Although the incidence and prevalence have generally reduced globally due to antibiotics and improved healthcare, it remains a significant public health concern in Brazil, echoing its prevalence in many developing nations around the world. RHD stands as a poignant testament to the intersection of socio-economic disparities and healthcare challenges within Brazil's diverse population. In Brazil, despite advancements in healthcare, RHD continues to impact communities, highlighting the urgent need for enhanced prevention strategies, access to quality healthcare services, and heightened awareness to combat this preventable, yet persistent, cardiac condition. Understanding the epidemiological landscape and socio-cultural factors influencing RHD in Brazil is crucial for developing targeted interventions aimed at mitigating its burden on individuals, families, and the healthcare system at large. Thus, our study focuses on analyzing age-related mortality rates linked to ARF and chronic RHD (ARHD) in Brazil from 2000 to 2021, particularly examining gender disparities.

Materials and methods

This retrospective cohort study employed a descriptive time-series approach, utilizing comprehensive nationwide data from Brazil spanning from 2000 to 2021 to assess trends in diverse age groups, among both sexes, enabling a detailed analysis of temporal patterns. Mortality data, extracted and categorized meticulously, were subjected to Joinpoint statistical analyses enabling comparative assessments, with average annual percent change (AAPC) and annual percent change (APC) serving as key metrics to quantify and interpret trends over the analyzed period.

Results

The acute RHD (ARHD)-related mortality declined over the analyzed years supported by AAPC, with higher mortality reduction in females. The age-adjusted mortality rate for "males and females" decreased from 78 to 67 deaths/100,000 from 2000 to 2021. Female mortality dropped from 85 to 69/100,000, and male mortality decreased from 73 to 63/100,000 over the same period. For ARHD, male age groups (20-29, 60-69, 70-79, 80+) showed declining mortality, while the 30-59 age group exhibited an upward. Females AAMR for chronic RHD (CRHD) decreased across all age groups, with significant reductions in the 80 years and above age group from 2000-2002 (APC: -11.94*) and steadily from 2002 onwards (APC: -1.33).

Conclusions

Our study revealed an overall decline in mortality rates for both acute and CRHD across both sexes. Females consistently exhibited higher mortality rates and a more pronounced reduction compared to males in both acute and CRHD. In ARHD, males experience the highest mortality in the 50-59 age group, while females have a peak in the 40-49 age group. The 60-69 age group had the highest mortality in CRHD for both sexes. Conversely, the 20-29 age group displayed the lowest mortality in CRHD, and the 80-89 age group had the lowest mortality in ARHD.

## Introduction

Rheumatic heart disease (RHD) is a chronic cardiovascular disorder with an infectious origin, contributing significantly to the disease burden in economically disadvantaged regions [1]. RHD begins with an acute rheumatic fever (ARF), which is a non-suppurative sequelae that occurs two to four weeks following group A Streptococcus (GAS) pharyngitis infection and may consist of arthritis, carditis, chorea, erythema marginatum, and subcutaneous nodules. Damage to cardiac valves may be chronic and progressive, resulting in cardiac decompensation [2].

Following GAS pharyngeal infection in susceptible individuals, which is known to be the leading cause of rheumatic fever, the immune response can trigger autoimmune reactions against host tissues, such as the valves most prone to the autoantibodies and progression to RHD, and the mitral and aortic valves [3]. Over several decades, the disease progression leads to valvular stenosis or regurgitation, resulting in structural cardiac abnormalities [4]. It is a major cause of preventable premature deaths and is a significant burden to the healthcare system [5], and the majority of ARF cases occur in low-middle-income countries.

A 2015 study encompassing 59 countries found that RHD contributes to approximately 0.6% of all deaths [6], and by 2019, RHD had affected 40.5 million individuals [7]. Annually, RHD gives rise to around 1,100,000 instances of heart failure (HF) and accounts for 320,000 deaths [8]. An estimated 15.6 million people are living with RHD, which accounts for 233,000 annual deaths and 282,000 newly reported cases annually. The broader ramifications of diseases related to GAS account for at least 517,000 deaths each year from severe GAS diseases and 663,000 new cases with 163,000 annual deaths from most invasive GAS diseases [9]. RHD is the leading cause of valvular heart disease in South American countries [10].

The areas most susceptible to RHD are those with a low socio-demographic index (SDI) [11]. In low-middle-income countries in the Southern Hemisphere and parts of Asia, ARF remains a significant contributor to premature mortality, with a particular impact on children aged 5-14 years [12]. In contrast, developed countries in the Northern Hemisphere, which saw the prevalence of the disease in the mid-20th century, are now free from new cases [13].

Globally, the incidence and prevalence of ARF and acute RHD (ARHD) have reduced in the late 20th century, and the decline is primarily due to the widespread use of benzathine penicillin G (BPG) to treat streptococcal pharyngitis, as well as improved living conditions and better healthcare access since these diseases are now associated with economic disadvantage and social inequality [14]. Additionally, due to increased awareness on the subject and the emergence of a better healthcare system in many parts of the world, the burden of the disease has fallen because its prevalence is inversely related to socioeconomic conditions [15].

The age pattern of RHD shows that incidence is more predominant in adolescence; the global peak incidence rate among females is 15-19 years, an,d among men, it is 10-14 years. However, the peak age group of the death rate in all SDI regions is at >85 years for both sexes from 1990 to 2019 [16]. The prevalence of the disease has been growing since the 20th century due to a lower number of cases reported from 1990 and the advent of antibiotics, echocardiography, and advances in research [17].

RHD has a low incidence in developed countries, with 0.1-0.4 cases per 1,000 school children in the US, while, in Brazil, these values are seven cases per 1,000 school children, showing that it is directly associated with environmental and socioeconomic factors [18]. The prevalence of RHD was 39 per 1,000 in adults from the Brazilian Amazon Basin, indicating the need for screening programs in remote areas [19].

Despite such a high disease burden, there have been very few studies that have recorded age-related mortality changes for ARHD and chronic RHD (CRHD), and so far no study has been conducted to identify the changes in mortality trends in Brazil. This gap is significant. As such, studies serve as a foundational basis for implementing interventions to alleviate the burden of disease at a national level. The aim of our study was to conduct an in-depth analysis of the age-related mortality rate, related to ARHD and CRHD in the male and female population of Brazil aged 20-80 years and above, spanning from 2000 to 2021, and find out the disparity in mortality trends between both sexes. Brazil stands out as an ideal setting for these investigations due to its diverse range of socioeconomic variations across different regions. The study comprises several components, including the analysis of ARHD age-adjusted mortality rates (AAMR) with consideration of gender from 2000 to 2021. Additionally, we explored the AAMR trends for ARHD across age groups ranging from 20 to 80 years and above, stratified by gender, during the same period. Furthermore, our investigation delves into the AAMR trends associated with CRHD, considering gender variations from 2000 to 2021. Lastly, we examine the AAMR trends for CRHD across age groups spanning from 20 to 80 years and above, based on gender, over the same time frame. This comprehensive approach aims to provide nuanced insights into the dynamics of RHD mortality in Brazil and contribute to informed interventions on a national scale.

## Materials and methods

Study design and data collection

We carried out a descriptive, time series study using RHD mortality data from Brazil, from the year 2000 to 2021. Mortality data were obtained from the Brazilian Hospital Information System (DATASUS ). Under the Sistema Único de Saúde (SUS), a Brazilian Unified Health System, DATASUS gathers information from all hospitalizations reimbursed by the SUS, which includes approximately 80% of the Brazilian population. The 10th edition of the International Classification of Disease (ICD 10) was used for records selection, and those coded E100 to E102 and E105 to E109 were included (which corresponds to ARHD and CRHD).

Statistical analysis

Population Estimates and Mortality Rates

Population and demographic information of counts based on variables, such as sex, age, and ARHD and CRHD, were obtained from the population estimates provided by the Brazilian Institute of Geography and Statistics (IBGE), under the Demographic and Socioeconomic Information section. We calculated the age-standardized mortality rate of RHD for ages 20-80 years and above. Mortality rates were expressed per 100,000 persons, and age-adjusted rates were calculated by direct standardization, using the Segi world standard population.

Time series analysis

To assess temporal trends in RHD mortality rates, we computed the average annual percentage changes (AAPCs), along with their respective 95% confidence intervals (CIs) employing joinpoint regression. The AAPC was derived as a geometrically weighted mean of different annual percentage change (APC) values acquired from the regression analysis. Joinpoint regression program (version 5.0.2., May 2023) is a trend analysis software developed by the Statistical Research and Applications Branch of the National Cancer Institute (Bethesda, MD), for the analysis of data from the Surveillance Epidemiology and End Results Program (SEER). Weighted Bayesian Information Criteria were used to test for the level of significance and determine the best-fitting combination of line segments and joinpoints [20]. This examination aimed to investigate variations in RHD mortality trends across categories such as age and sex.

## Results

ARHD mortality based on sex

**Figure 1:**
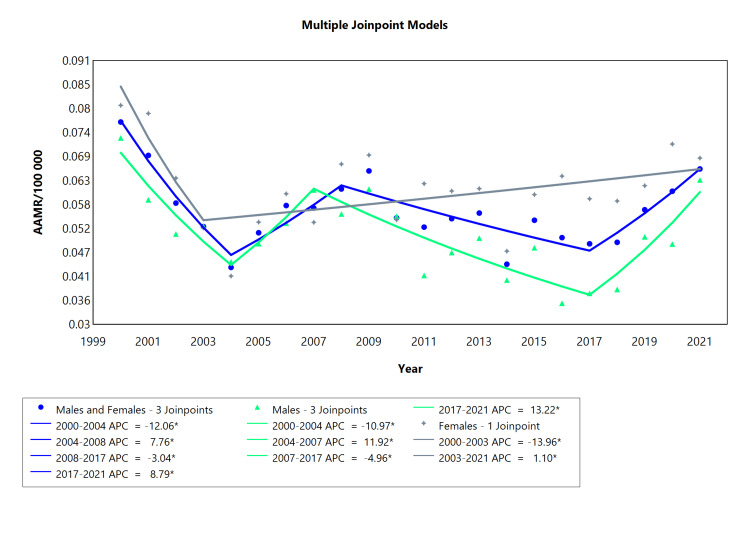
AAMR trend joinpoint analysis of acute rheumatic heart disease for the variable sex during the period 2000-2021. *Significant at P < 0.05 level AAMR = Age-adjusted mortality rate; APC = Annual percent change

With regards to both sexes for ARHD, the trend was downward for the categories of males, females, and males and females combined, over the analyzed years, as demonstrated by the AAPC (Table 1, Appendix Table 7). The combined male/female and males (2000-2021) had four segments, the female only had two segments, and males had four segments, as shown in Table 1 (Appendix Table 7), and visually represented in Figure 1. In addition, the reduction in mortality was more pronounced among females than males (see AAPC values). The AAMR between 2000 and 2021 for the combined category of "males and females," ranged from 78 deaths per 100,000 inhabitants in 2000 to 67 deaths per 100,000 in 2021 (see Figure 1). In 2000, there were 85 fatalities per 100,000 people in the female population, and by 2021 the number dropped to 69 deaths per 100,000 people. On the other hand, the initial AAMR for males was 73 fatalities per 100,000 in 2000, and by 2021, it decreased to 63 deaths per 100,000. This overall trend underscores a positive shift in mortality rates over the analyzed years.

**Table 1:** AAMR trend joinpoint analysis of acute rheumatic heart disease for the variable sex during the period 2000-2021. *Significant at P < 0.05 level AAMR = Age-adjusted mortality rate; AAPC = Average annual percent change; APC = Annual percent change

Cohort	Period (year)	APC (with CI)	AAPC (with CI)	Interpretation
Males and Females	2000-2004	-12.06* (-18.07 to -8.33)	-0.74* (-1.35 to -0.12)	Decrease
	2004-2008	7.76* (2.77 to 14.53)		Increase
	2008-2017	-3.04* (-6.88 to -1.76)		Decrease
	2017-2021	8.79* (3.98 to 19.50)		Increase
Males	2000-2004	-10.97* (-23.89 to -4.63)	-0.66 (-1.81 to 0.42)	Decrease
	2004-2007	11.92* (1.22 to 20.00)		Increase
	2007-2017	-4.96* (-15.07 to -3.34)		Decrease
	2017-2021	13.22* (3.75 to 35.08)		Increase
Females	2000-2003	-13.96* (-26.09 to -2.79)	-1.20 (-2.07 to 0.12)	Decrease
	2003-2021	1.10* (0.14 to 3.04)		Increase

Age-specific ARHD-related mortality in males

**Figure 2:**
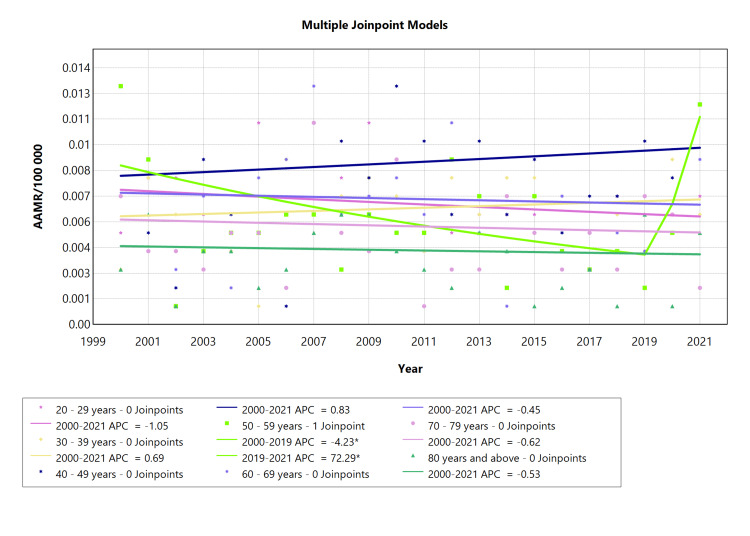
AAMR trend joinpoint analysis of acute rheumatic heart disease for the variable age for males during the period 2000-2021. *Significant at P < 0.05 level AAMR = Age-adjusted mortality rate; APC = Annual percent change

The analysis of AAMR of ARHD in male age groups showed that those aged 20-29 years, 60-69 years, 70-79 years, and 80 years and above experienced an overall declining mortality from 2000 to 2021 (Figure 2). Males in the 30-39 and 40-49 age groups exhibited a considerably more consistent upward trend over time (Table 2, Appendix Table 8 ). From 2001 to 2019, the 50-59 age group experienced a trend that was marked by a notable fall (APC: -4.23), but between 2019 and 2021, the death rate spiked with the highest mortality rate for this cohort (APC: 72.29) occurring in just three years. A more pronounced decline occurred in cohorts 60-69 years. The mortality rate was lower in the youngest (below 29 years) and oldest age groups (above 60 years) showing negative APC in these extremes. This study highlighted that, in comparison to younger (below 29 years) and older age groups (above 60 years), mortality was higher in cohorts comprising the intermediate age group (30-59 years), as illustrated in Figure 2.

**Table 2:** AAMR trend joinpoint analysis of acute rheumatic heart disease for the variable age for males during the period 2000-2021. *Significant at P < 0.05 level AAMR = Age-adjusted mortality rate; AAPC = Average annual percent change; APC = Annual percent change

Cohort	Period (year)	APC (with CI)	AAPC (with CI)	Interpretation
20-29 years	2000-2021	-1.04 (-3.54 to 1.43)	-1.04 (-3.54 to 1.43)	Decrease
30-39 years	2000-2021	0.69 (-2.72 to 4.38)	0.69 (-2.72 to 4.38)	Increase
40-49 years	2000-2021	0.83 (-2.27 to 4.30)	0.83 (-2.27 to 4.30)	Increase
50-59 years	2000-2019	-4.23* (-18.80 to -1.40)	1.28 (-2.94 to 4.30)	Decrease
	2019-2021	72.29* (2.36 to 149.01)		Increase
60-69 years	2000-2021	-0.45 (-4.11 to 3.13)	-0.45 (-4.11 to 3.13)	Decrease
70-79 years	2000-2021	-0.62 (-4.43 to 3.17)	-0.62 (-4.43 to 3.17)	Decrease
80 years and above	2000-2021	-0.53 (-5.60 to 4.45)	-0.53 (-5.60 to 4.45)	Decrease

Age-specific ARHD-related mortality in females

**Figure 3:**
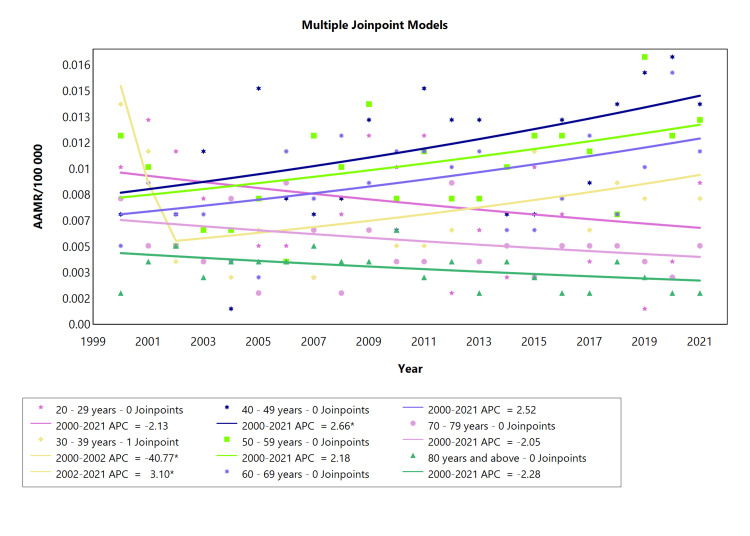
AAMR trend joinpoint analysis of acute rheumatic heart disease for the variable age for females during the period 2000-2021. *Significant at P < 0.05 level AAMR = Age-adjusted mortality rate; APC = Annual percent change

Analyzing the temporal trends in APC for ARHD among the female cohort from 2000 to 2021 showed that the age cohort of 20-29 years exhibited a relatively stable trend with a slight decline in ARHD-related mortality from 2000 to 2021 (APC: -2.13, see Figure 3). The age cohort of 30-39 showed a notable initial decline from 2000 to 2002 (APC: -40.8; see Table 3, Appendix Table 9), followed by a more recent increase, which is quite significant (2002-2021). Cohorts comprising 50-59, 60-69, 70-79, and 80 and above showed a relatively stable ARHD-related mortality with only minor fluctuations. Distinctively, these fluctuations exhibit an upward trend for age cohorts of 50-59 and 60-69, while cohorts comprising 70-79 and 80 and above show a downward trajectory.

**Table 3:** AAMR trend joinpoint analysis of acute rheumatic heart disease for the variable age for females during the period 2000-2021. *Significant at P < 0.05 level AAMR = Age-adjusted mortality rate; AAPC = Average annual percent change; APC = Annual percent change

Cohort	Period (year)	APC (with CI)	AAPC (with CI)	Interpretation
20-29 years	2000-2021	-2.13 (-7.00 to 2.39)	-2.13 (-7.00 to 2.39)	Decrease
30-39 years	2000-2002	-40.8* (-56.9 to -3.26)	-2.20 (-4.68 to 1.72)	Decrease
	2002-2021	3.10* (0.58 to 13.7)		Increase
40-49 years	2000-2021	2.66* (0.32 to 5.57)	2.66* (0.32 to 5.57	Increase
50-59 years	2000-2021	2.18 (-0.14 to 4.95)	2.18 (-0.14 to 4.95)	Increase
60-69 years	2000-2021	2.52 (-0.11 to 5.64)	2.52 (-0.11 to 5.64)	Increase
70-79 years	2000-2021	-2.05 (-4.83 to 0.55)	-2.05 (-4.83 to 0.55)	Decrease
80 years and above	2000-2021	-2.28 (-5.06 to 0.26	-2.28 (-5.06 to 0.26	Decrease

CRHD-related mortality based on sex

**Figure 4:**
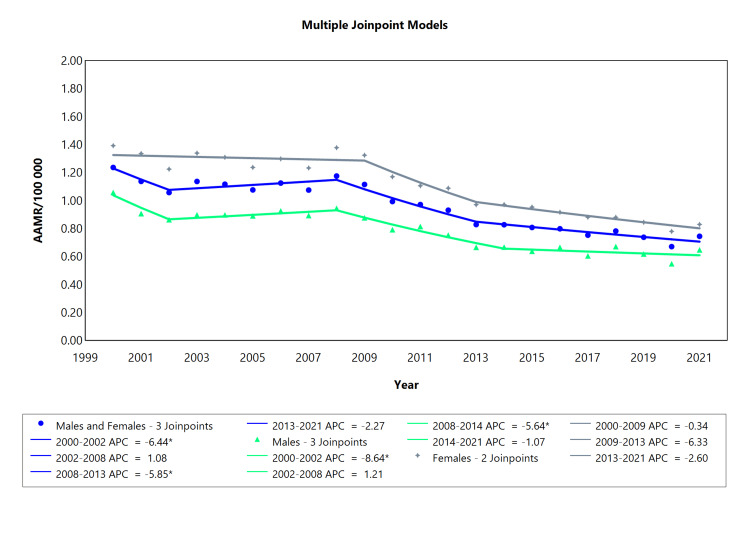
AAMR trend joinpoint analysis of chronic rheumatic heart disease for the variable sex during the period 2000-2021. *Significant at P < 0.05 level AAMR = Age-adjusted mortality rate; APC = Annual percent change

The AAMR for Chronic RHD between 2000 and 2021 for the category “males and females” ranged from 13 deaths/100,000 to eight deaths/100,000 inhabitants (Figure 4). Females exhibited a higher mortality rate (14 deaths/100,000 inhabitants in 2000, declining to nine deaths/100,000 in 2021) compared to males, who had a mortality rate of 11 deaths/100,000 in 2000, decreasing to nine deaths/100,000 in 2021. The chronic RHD mortality rate trend was downward for the categories of males, females, and males and females combined, over the analyzed years, as demonstrated by the average annual percent change (AAPC, Figure 4). The female population had three segments (2000-2009, 2009-2013, and 2013-2021), with a decline in all segments. In contrast, there were four notable APC segments in the male population: a rapid decline from 2000 to 2002 (APC: -5.63), a steady rise from 2002 to 2008, a strong decrease from 2008 to 2014 (APC: -5.64), and a gradual fall from 2014 to 2021 (APC: -1.07; see Figure 4). Similarly, a combined male and female population had four segments with significant APC per segment in both populations, as shown in Table 4 (Appendix Table 10) and visually represented in Figure 4. In addition, the reduction in mortality was more pronounced among females and the combined male and female population as compared to males alone (see AAPC values).

**Table 4:** AAMR trend joinpoint analysis of chronic rheumatic heart disease for the variable sex during the period 2000-2021. *Significant at P < 0.05 level AAMR = Age-adjusted mortality rate; AAPC = Average annual percent change; APC = Annual percent change

Cohort	Period (year)	APC (with CI)	AAPC (with CI)	Interpretation
Males and Females	2000-2002	-6.44* (-10.3 to -0.07)	-2.60* (-2.98 to -2.09)	Decrease
	2002-2008	1.08 (-6.49 to 5.61)		Increase
	2008-2013	-5.85* (-9.22 to -0.73)		Decrease
	2013-2021	-2.27 (-4.97 to 2.43)		Decrease
Males	2000-2002	-8.64* (-12.6 to -2.28)	-2.50* (-2.89 to -1.99)	Decrease
	2002-2008	1.21 (-0.41 to 5.53)		Increase
	2008-2014	-5.64* (-9.53 to -3.80)		Decrease
	2014-2021	-1.07 (-2.61 to 3.39)		Decrease
Females	2000-2009	-0.34 (-2.38 to 1.08)	-2.36* (-2.77 to -1.96)	Decrease
	2009-2013	-6.33 (-9.20 to 1.26)		Decrease
	2013-2021	-2.60 (-4.00 to 1.82)		Decrease

Age-specific CRHD-related mortality in males

**Figure 5:**
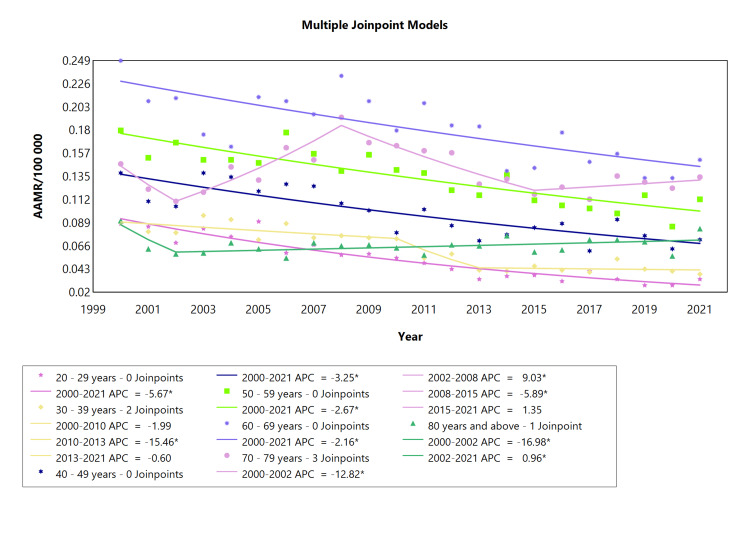
AAMR trend joinpoint analysis of chronic rheumatic heart disease for the variable age for males during the period 2000-2021. *Significant at P < 0.05 level AAMR = Age-adjusted mortality rate; APC = Annual percent change

An overall falling tendency was found when the AAMR for CRHD in males was examined across different age ranges. The age groups 20-29, 40-49, 50-59, and 60-69 years showed a steady decline in mortality (APC: -5.67, -3.25, -2.67, and -2.16, respectively; see Table 5, Appendix Table 11). The cohort comprising 30-39 years displayed a significant decline in mortality amidst two relatively minor downward trends. In the age range of 70-79 years, fluctuations were more pronounced with an abrupt and significant decline from 2000 to 2002 (APC: -12.8), followed by a strong rise from 2002 to 2008 (APC: 9.08), then a decline from 2008 to 2015 (APC: -5.89), and finally a gradual increase from 2015 to 2021, as seen in Figure 5. The 80-year-old cohort experienced the largest fall in AAMR between 2000 and 2002 (APC: -16.98). From 2002 onwards, there was a consistent upward trend in AAMR.

**Table 5:** AAMR trend joinpoint analysis of chronic rheumatic heart disease for the variable age for males during the period 2000-2021. *Significant at P < 0.05 level AAMR = Age-adjusted mortality rate; AAPC = Average annual percent change; APC = Annual percent change

Cohort	Period (year)	APC (with CI)	AAPC (with CI)	Interpretation
20-29 years	2000-2021	-5.67* (-6.82 to -4.77)	-5.67* (-6.82 to -4.77)	Decrease
30-39 years	2000-2010	-1.99 (-3.94 to - 2.84)	-3.52* (-4.55 to -2.34)	Decrease
	2010-2013	-15.4* (-20.6 to -5.53)		Decrease
	2013-2021	-0.60 (-4.66 to 12.9)		Decrease
40-49 years	2000-2021	-3.25* (-4.58 to -2.08)	-3.2470* (-4.58 to - 2.08)	Decrease
50-59 years	2000-2021	-2.67* (-3.47 to -1.94)	-2.67* (-3.47 to -1.94)	Decrease
60-69 years	2000-2021	-2.16* (-3.19 to -1.20)	-2.16* (-3.19 to -1.20)	Decrease
70-79 years	2000-2002	-12.8* (-19.8 to -0.44)	-0.48 (-1.17 to 0.51)	Decrease
	2002-2008	9.03* (6.14 to 17.7)		Increase
	2008-2015	-5.89* (-11.8 to -3.71)		Decrease
	2015-2021	1.355 (-1.58 to 10.7)		Increase
80 Years and above	2000-2002	-17.0* (-23.6 to -2.10)	-0.91 (-1.65 to 0.28)	Decrease
	2002-2021	0.96* (0.22 to 2.41)		Increase

Age-specific CRHD-related mortality in females

**Figure 6:**
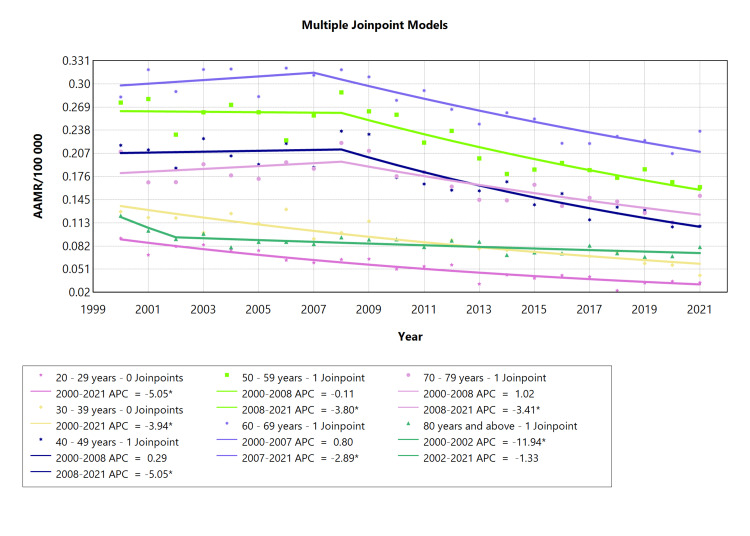
AAMR trend joinpoint analysis of chronic rheumatic heart disease for the variable age for females during the period 2000-2021. *Significant at P < 0.05 level AAMR = Age-adjusted mortality rate; APC = Annual percent change

Examining the AAMR for females across various age ranges for chronic RHD revealed an overall declining pattern. The most substantial reduction occurred in the 80-year and above age group from 2000 to 2002 (APC: -11.9*, whereas from 2002 onwards, there was a steady decline (APC: -1.33; see Table 6, Appendix Table 12). The smallest decline was observed in the 50-59 year age group spanning from 2000 to 2008 (APC: -0.11, Figure 6). The AAMR in the 20-29 year and 30-39 age group experienced a similar pattern of a significant decrease in mortality rates from 2001 to 2021, characterized by a downward slope (APC: -5.05* and -3.94*, respectively; see Figure 6).

**Table 6:** AAMR trend joinpoint analysis of chronic rheumatic heart disease for the variable age for females during the period 2000-2021. *Significant at P < 0.05 level AAMR = Age-adjusted mortality rate; AAPC = Average annual percent change; APC = Annual percent change

Cohort	Period (year)	APC (with CI)	AAPC (with CI)	Interpretation
20-29 years	2000-2021	-5.05* (-6.05 to -4.20)	-5.05* (-6.05 to -4.20)	Decrease
30-39 years	2000-2021	-3.94* (-4.80 to -3.18)	-3.94* (-4.80 to -3.18)	Decrease
40-49 years	2000-2008	0.28 (-2.56 to 17.7)	-3.05* (-5.29 to -1.28)	Increase
	2008-2021	-5.05* (-15.7 to -3.63)		Decrease
50-59 years	2000-2008	-0.11 (-2.03 to 9.43)	-2.41* (-3.50 to -1.48)	Decrease
	2008-2021	-3.80* (-9.31 to -2.81)		Decrease
60-69 years	2000-2007	0.80 (-1.16 to 7.52)	-1.68* (-2.38 to -0.90)	Increase
	2007-2021	-2.89* (-5.00 to -2.18)		Decrease
70-79 years	2000-2008	1.02 (-1.54 to 14.8)	-1.75* (-3.32 to -0.28)	Increase
	2008-2021	-3.41* (-12.5 to -2.11)		Decrease
80 years and above	2000-2002	-11.9* (-17.9 to -1.25)	-2.40* (-3.14 to -1.18)	Decrease
	2002-2021	-1.33 (-7.69 to 4.06)		Decrease

## Discussion

ARHD-related mortality based on sex and age

Our study found that the mortality rate of acute RHD has been declining since 2000 in both the male and female populations, although females exhibited an overall higher mortality rate. These sex disparities are attributed to the often vague presentation of symptoms among females, leading to delayed recognition of dyspnea and other cardiac symptoms [21]. However, other studies found no specific variation in the number and severity of cardiac symptoms between men and women, except for the fact that females are less likely to report their symptoms [21]. This divergence in mortality trends can also be attributed to the autoimmune response, which may vary between the sexes due to differences in severity triggered by group A beta-hemolytic Streptococcus pharyngitis [22]. Studies also reveal that both ARHD and CRHD indicators, such as DALYs and mortality rates, demonstrate higher figures within the female population, particularly among women of childbearing age when compared to their male counterparts [3]. One reason behind the higher mortality rates among middle-aged women could be due to pregnancy, since pregnant women experience higher straining forces on the heart due to the growing fetus and turbulent hemodynamic flow along with an overall higher risk of thromboembolic events [23]. Additionally, this trend has been associated with factors such as higher fertility rates in low-income countries, which result from a combination of factors, including a lack of awareness, socioeconomic constraints, and limited access to timely healthcare facilities [3]. These factors can collectively damage the valves further and contribute to a higher mortality trend in women with a history of RHD. In a study involving 50 hospitalized pregnant patients with heart disease in Senegal, 46 of them had RHD, and the maternal mortality rate was 34% [24].

The analysis of APC for ARHD in the male cohort from 2000 to 2021 reveals intricate insights into the shifting mortality trends of ARHD among males (see APC in Table 2). The mortality rate in males aged 20-29 years for ARHD had a slight decrease (APC: -1.04) over the entire study period from 2000 to 2021. The declines observed in younger age groups can be ascribed to the advancements in health resources and preventive and control strategies that have been put into place during the last few decades [25]. There has been a significant decreasing trend in age-standardized mortality rates, disability-adjusted life years (DALYs), and years of life lost (YLLs) for RHD in the Asian region from 1990 to 2019 and globally [26]. Male cohorts in the 30-39 years and in 40-49 years displayed a minor increase in ARHD-related mortality, and this is in line with the results from a study that showed that late adolescence and early adulthood (somewhat corresponding to our cohort of interest) are mostly at risk of death from RHD, possibly due to the drop-out from health care, and gaps in adolescent health care services in these age groups, particularly in middle- and low-income countries [27]. This finding would otherwise have seemed counterintuitive in light of other studies, which showed that while the RHD burden has increased mildly in terms of age-standardized prevalence rates (ASPR), it has significantly decreased in terms of age-standardized mortality rates (ASMR) and age-standardized morbidity rates (AMDR) from 1990 to 2019 [28]. The only significant fluctuation in the mortality trend was seen in males aged 50-59 years. During 2000-2019, this age group showed a significant decline in ARHD-related mortality (APC: -4.23), which was followed by an abrupt increase in mortality rates with a drastic spike (APC: 72.3; see Table 2). Males in the 60-69 years, 70-79 years, and 80 years and above exhibited relatively stable ARHD-related mortality, as the APC values for these groups were low (around zero).

Essentially, this analysis reveals almost similar and mostly consistent trends in ARHD-related mortality among males of different age groups. The decreasing trend is obvious in the 20-29, 60-69, 70-79, and 80-year and above cohorts. It is only the 50-59-year-old cohort that shows a decrease, followed by a dramatic increase in the last two years, and the increase in recent years demands urgent attention and further research to address the concerning rise in male ARHD mortality rates. This analysis underscores the importance of tailored strategies to tackle this issue among the male population in Brazil.

The decrease in ARHD-related mortality rate in females through 2000-2003 matches the trend observed in males through 2000-2004. For females in the age range of 20-29 (2000-2021), there was a decline in mortality rates. However, in the 30-39 age group, a distinct pattern emerged, characterized by a sharp decline (2000-2002) and a subsequent steady rise from 2002 to 2021. The higher rates of RHD in the female population aged 20 and above could be linked to increased detection rates among females during their reproductive years, often as part of antenatal care [29].

CRHD-related mortality based on sex and age

CRHD-related mortality was higher in females than in males (Figure 4). Higher AAMR in females is a function of RHD predominance in females [30]. A notable sex disparity persists in mitral valve pathology, with females exhibiting a higher prevalence of RHD compared to males [31]. Among patients with RHD, mitral stenosis stands out as the most prevalent heart lesion [32], which is important because the higher mortality rate in females could also be explained by significantly more frequent involvement of the mitral valve in females versus in males, in whom aortic valve involvement is more frequent [33]. In general, the trajectory for CRHD mortality in both males and females headed in the same direction, which is downwards, although females displayed a steeper decline. Mortality rates for CRHD in males showed a consistent decline in most age ranges (20-29, 40-49, 50-59, and 60-69). However, fluctuations and significant changes were observed in specific cohorts, such as an abrupt decline followed by a steep rise in the 70-79 years group and a consistent upward trend in the 80-year-old and above cohort after a sharp initial decline. Despite having a declining trajectory, the cohort comprising 60-69-year-old males maintained the highest mortality rate throughout the study, while the youngest cohort comprising 20-29-year-old age exhibited the lowest mortality rate. Mortality rates in different female age groups with CRHD varied over the analyzed period.

Sex-based disparities in CRHD and ARHD mortality patterns

In our study, when comparing the mortality rates of ARHD and CRHD, we observed differences in the trends based on sex over the analyzed period. ARHD-related mortality for males and females combined showed a downward trend in general, though the decline was interrupted at multiple joinpoints with rises. A relatively smoother decline was observed for CRHD-related mortality for males and females combined, but the reduction was more significant among females and the combined male and female population compared to males alone (Figure 4). It is pertinent to note that the reduction in mortality was more pronounced in females than in males in both ARHD and CRHD. These subtle differences and similarities in mortality trends between ARHD and CRHD may be attributed to various factors related to the nature and progression of the disease in each sex, but the discussion on such is beyond the scope of this article and remains a gap for further studies.

Limitations and strengths of the study

In spite of the limitation regarding the exclusion of pediatric and adolescent age cohorts as subjects of investigation, our study distinguishes itself by examining RHD mortality within age-specific cohorts while highlighting sex disparities. This perspective enhances the depth and comprehensiveness of our research, contributing valuable insights to the understanding of RHD mortality across diverse age groups and sex categories. Additionally, the unique contribution of our study includes but is not limited to highlighting the fluctuations in mortality rates in acute RHD, emphasizing the need for continuous monitoring and adjustment of healthcare strategies. The identification of age groups with the highest and lowest mortality rates in ARHD and CRHD allows for better targeting of preventive measures and healthcare resources. Our study also uniquely showed the sharp increase in mortality rates among males aged 50-59 in ARHD, underscoring the necessity for focused interventions in this specific demographic. Lastly, the consistently higher mortality rates in females for both acute and chronic RHD together with a more pronounced reduction in mortality for females in both ARHD and CRHD suggests a possible sex-based response to treatment and reflects the importance of personalized care. It is important to acknowledge that the precise reasons behind the more pronounced decline in mortality rates among females compared to males were not within the purview of this study. Exploring the factors contributing to this sex-specific variation remains an area for future research. The study has also not delved into the underlying causes for the increasing mortality in acute RHD among males and the concurrent decrease in CRHD. These complexities warrant further investigation in future studies to unveil the underlying factors responsible for this observed trend.

## Conclusions

Our study found an overall decreasing trajectory for male and female mortality rates for both ARHD and CRHD. Although females consistently exhibit elevated mortality rates in both ARHD and CRHD, the reduction in mortality was more pronounced in females than in males in both ARHD and CRHD. The decline was relatively consistent for both sexes for CRHD-related mortality while marked with fluctuations in ARHD-related mortality, characterized by significant declines and subsequent increases. Males aged 50-59 years showed a significant decline in ARHD-related mortality, but the last three years of the study marked a sharp increase in mortality rates in this cohort. In ARHD, the highest mortality for males was recorded in the 50-59 age group, while, for females, it occurred in the 40-49 age group. In CRHD, the 60-69 age group records the highest mortality for both sexes. Notably, the cohort in ARHD with the highest mortality rate exhibits an increasing trend among both sexes, whereas, in CRHD, the highest mortality rate cohort displays a decreasing trend. Among both sexes, the lowest mortality rates in CRHD are observed in the 20-29 age cohort, while, in ARHD, the lowest mortality occurs in the 80-89 age group.

## Appendices

**Table 7:** AAMR trend joint point analysis of acute rheumatic heart disease for the variable sex during the period 2000-2021.

Sex	Year	AAMR/100,000
Males and Females	2000	0.08
Males and Females	2001	0.07
Males and Females	2002	0.06
Males and Females	2003	0.05
Males and Females	2004	0.04
Males and Females	2005	0.05
Males and Females	2006	0.06
Males and Females	2007	0.06
Males and Females	2008	0.06
Males and Females	2009	0.07
Males and Females	2010	0.05
Males and Females	2011	0.05
Males and Females	2012	0.05
Males and Females	2013	0.06
Males and Females	2014	0.04
Males and Females	2015	0.05
Males and Females	2016	0.05
Males and Females	2017	0.05
Males and Females	2018	0.05
Males and Females	2019	0.06
Males and Females	2020	0.06
Males and Females	2021	0.07
Males	2000	0.07
Males	2001	0.06
Males	2002	0.05
Males	2003	0.05
Males	2004	0.04
Males	2005	0.05
Males	2006	0.05
Males	2007	0.06
Males	2008	0.06
Males	2009	0.06
Males	2010	0.05
Males	2011	0.04
Males	2012	0.05
Males	2013	0.05
Males	2014	0.04
Males	2015	0.05
Males	2016	0.03
Males	2017	0.04
Males	2018	0.04
Males	2019	0.05
Males	2020	0.05
Males	2021	0.06
Females	2000	0.08
Females	2001	0.08
Females	2002	0.06
Females	2003	0.05
Females	2004	0.04
Females	2005	0.05
Females	2006	0.06
Females	2007	0.05
Females	2008	0.07
Females	2009	0.07
Females	2010	0.05
Females	2011	0.06
Females	2012	0.06
Females	2013	0.06
Females	2014	0.05
Females	2015	0.06
Females	2016	0.06
Females	2017	0.06
Females	2018	0.06
Females	2019	0.06
Females	2020	0.07
Females	2021	0.07

**Table 8:** AAMR trend joinpoint analysis of acute rheumatic heart disease for the variable age for males during the period 2000-2021.

Age range	Year	AAMR/100,000
0-4 years	2000	0.00
0-4 years	2001	0.00
0-4 years	2002	0.00
0-4 years	2003	0.00
0-4 years	2004	0.00
0-4 years	2005	0.00
0-4 years	2006	0.00
0-4 years	2007	0.00
0-4 years	2008	0.00
0-4 years	2009	0.00
0-4 years	2010	0.00
0-4 years	2011	0.00
0-4 years	2012	0.00
0-4 years	2013	0.00
0-4 years	2014	0.00
0-4 years	2015	0.00
0-4 years	2016	0.00
0-4 years	2017	0.00
0-4 years	2018	0.00
0-4 years	2019	0.00
0-4 years	2020	0.00
0-4 years	2021	0.00
5-9 years	2000	0.00
5-9 years	2001	0.00
5-9 years	2002	0.00
5-9 years	2003	0.00
5-9 years	2004	0.00
5-9 years	2005	0.00
5-9 years	2006	0.00
5-9 years	2007	0.00
5-9 years	2008	0.00
5-9 years	2009	0.00
5-9 years	2010	0.00
5-9 years	2011	0.00
5-9 years	2012	0.00
5-9 years	2013	0.00
5-9 years	2014	0.00
5-9 years	2015	0.00
5-9 years	2016	0.00
5-9 years	2017	0.00
5-9 years	2018	0.00
5-9 years	2019	0.00
5-9 years	2020	0.00
5-9 years	2021	0.00
10-14 years	2000	0.01
10-14 years	2001	0.01
10-14 years	2002	0.01
10-14 years	2003	0.01
10-14 years	2004	0.01
10-14 years	2005	0.01
10-14 years	2006	0.00
10-14 years	2007	0.00
10-14 years	2008	0.00
10-14 years	2009	0.00
10-14 years	2010	0.00
10-14 years	2011	0.00
10-14 years	2012	0.00
10-14 years	2013	0.00
10-14 years	2014	0.00
10-14 years	2015	0.00
10-14 years	2016	0.00
10-14 years	2017	0.00
10-14 years	2018	0.00
10-14 years	2019	0.00
10-14 years	2020	0.00
10-14 years	2021	0.00
15-19 years	2000	0.01
15-19 years	2001	0.00
15-19 years	2002	0.01
15-19 years	2003	0.00
15-19 years	2004	0.00
15-19 years	2005	0.00
15-19 years	2006	0.01
15-19 years	2007	0.00
15-19 years	2008	0.01
15-19 years	2009	0.01
15-19 years	2010	0.00
15-19 years	2011	0.00
15-19 years	2012	0.00
15-19 years	2013	0.01
15-19 years	2014	0.00
15-19 years	2015	0.00
15-19 years	2016	0.00
15-19 years	2017	0.00
15-19 years	2018	0.00
15-19 years	2019	0.01
15-19 years	2020	0.00
15-19 years	2021	0.00
20-29 years	2000	0.01
20-29 years	2001	0.01
20-29 years	2002	0.01
20-29 years	2003	0.01
20-29 years	2004	0.00
20-29 years	2005	0.01
20-29 years	2006	0.01
20-29 years	2007	0.01
20-29 years	2008	0.01
20-29 years	2009	0.01
20-29 years	2010	0.00
20-29 years	2011	0.00
20-29 years	2012	0.00
20-29 years	2013	0.01
20-29 years	2014	0.01
20-29 years	2015	0.01
20-29 years	2016	0.00
20-29 years	2017	0.00
20-29 years	2018	0.01
20-29 years	2019	0.01
20-29 years	2020	0.01
20-29 years	2021	0.01
30-39 years	2000	0.00
30-39 years	2001	0.01
30-39 years	2002	0.01
30-39 years	2003	0.00
30-39 years	2004	0.00
30-39 years	2005	0.00
30-39 years	2006	0.01
30-39 years	2007	0.01
30-39 years	2008	0.01
30-39 years	2009	0.01
30-39 years	2010	0.01
30-39 years	2011	0.00
30-39 years	2012	0.01
30-39 years	2013	0.01
30-39 years	2014	0.01
30-39 years	2015	0.01
30-39 years	2016	0.00
30-39 years	2017	0.01
30-39 years	2018	0.01
30-39 years	2019	0.00
30-39 years	2020	0.01
30-39 years	2021	0.01
40-49 years	2000	0.01
40-49 years	2001	0.00
40-49 years	2002	0.00
40-49 years	2003	0.01
40-49 years	2004	0.01
40-49 years	2005	0.01
40-49 years	2006	0.00
40-49 years	2007	0.01
40-49 years	2008	0.01
40-49 years	2009	0.01
40-49 years	2010	0.01
40-49 years	2011	0.01
40-49 years	2012	0.01
40-49 years	2013	0.01
40-49 years	2014	0.01
40-49 years	2015	0.01
40-49 years	2016	0.01
40-49 years	2017	0.01
40-49 years	2018	0.01
40-49 years	2019	0.01
40-49 years	2020	0.01
40-49 years	2021	0.01
50-59 years	2000	0.01
50-59 years	2001	0.01
50-59 years	2002	0.00
50-59 years	2003	0.00
50-59 years	2004	0.01
50-59 years	2005	0.00
50-59 years	2006	0.01
50-59 years	2007	0.01
50-59 years	2008	0.00
50-59 years	2009	0.01
50-59 years	2010	0.01
50-59 years	2011	0.00
50-59 years	2012	0.01
50-59 years	2013	0.01
50-59 years	2014	0.00
50-59 years	2015	0.01
50-59 years	2016	0.00
50-59 years	2017	0.00
50-59 years	2018	0.00
50-59 years	2019	0.00
50-59 years	2020	0.00
50-59 years	2021	0.01
60-69 years	2000	0.01
60-69 years	2001	0.00
60-69 years	2002	0.00
60-69 years	2003	0.01
60-69 years	2004	0.00
60-69 years	2005	0.01
60-69 years	2006	0.01
60-69 years	2007	0.01
60-69 years	2008	0.01
60-69 years	2009	0.01
60-69 years	2010	0.01
60-69 years	2011	0.01
60-69 years	2012	0.01
60-69 years	2013	0.00
60-69 years	2014	0.00
60-69 years	2015	0.01
60-69 years	2016	0.01
60-69 years	2017	0.00
60-69 years	2018	0.00
60-69 years	2019	0.00
60-69 years	2020	0.01
60-69 years	2021	0.01
70-79 years	2000	0.01
70-79 years	2001	0.00
70-79 years	2002	0.00
70-79 years	2003	0.00
70-79 years	2004	0.01
70-79 years	2005	0.01
70-79 years	2006	0.00
70-79 years	2007	0.01
70-79 years	2008	0.00
70-79 years	2009	0.00
70-79 years	2010	0.01
70-79 years	2011	0.00
70-79 years	2012	0.00
70-79 years	2013	0.00
70-79 years	2014	0.01
70-79 years	2015	0.00
70-79 years	2016	0.00
70-79 years	2017	0.01
70-79 years	2018	0.00
70-79 years	2019	0.01
70-79 years	2020	0.01
70-79 years	2021	0.00
80 years and above	2000	0.00
80 years and above	2001	0.01
80 years and above	2002	0.00
80 years and above	2003	0.00
80 years and above	2004	0.00
80 years and above	2005	0.00
80 years and above	2006	0.00
80 years and above	2007	0.01
80 years and above	2008	0.01
80 years and above	2009	0.01
80 years and above	2010	0.00
80 years and above	2011	0.00
80 years and above	2012	0.00
80 years and above	2013	0.00
80 years and above	2014	0.00
80 years and above	2015	0.00
80 years and above	2016	0.00
80 years and above	2017	0.00
80 years and above	2018	0.00
80 years and above	2019	0.01
80 years and above	2020	0.00
80 years and above	2021	0.00

**Table 9:** AAMR trend joinpoint analysis of acute rheumatic heart disease for the variable age for females during the period 2000-2021.

Age range	Year	AAMR/100,000
0-4 years	2000	0.00
0-4 years	2001	0.00
0-4 years	2002	0.00
0-4 years	2003	0.00
0-4 years	2004	0.00
0-4 years	2005	0.00
0-4 years	2006	0.00
0-4 years	2007	0.00
0-4 years	2008	0.00
0-4 years	2009	0.00
0-4 years	2010	0.00
0-4 years	2011	0.00
0-4 years	2012	0.00
0-4 years	2013	0.00
0-4 years	2014	0.00
0-4 years	2015	0.00
0-4 years	2016	0.00
0-4 years	2017	0.00
0-4 years	2018	0.00
0-4 years	2019	0.00
0-4 years	2020	0.00
0-4 years	2021	0.00
5-9 years	2000	0.00
5-9 years	2001	0.00
5-9 years	2002	0.00
5-9 years	2003	0.00
5-9 years	2004	0.00
5-9 years	2005	0.00
5-9 years	2006	0.01
5-9 years	2007	0.00
5-9 years	2008	0.00
5-9 years	2009	0.00
5-9 years	2010	0.00
5-9 years	2011	0.00
5-9 years	2012	0.00
5-9 years	2013	0.00
5-9 years	2014	0.00
5-9 years	2015	0.00
5-9 years	2016	0.00
5-9 years	2017	0.00
5-9 years	2018	0.00
5-9 years	2019	0.00
5-9 years	2020	0.00
5-9 years	2021	0.00
10-14 years	2000	0.01
10-14 years	2001	0.01
10-14 years	2002	0.01
10-14 years	2003	0.01
10-14 years	2004	0.00
10-14 years	2005	0.00
10-14 years	2006	0.00
10-14 years	2007	0.00
10-14 years	2008	0.00
10-14 years	2009	0.00
10-14 years	2010	0.00
10-14 years	2011	0.00
10-14 years	2012	0.00
10-14 years	2013	0.00
10-14 years	2014	0.00
10-14 years	2015	0.00
10-14 years	2016	0.00
10-14 years	2017	0.00
10-14 years	2018	0.00
10-14 years	2019	0.00
10-14 years	2020	0.00
10-14 years	2021	0.01
15-19 years	2000	0.01
15-19 years	2001	0.00
15-19 years	2002	0.01
15-19 years	2003	0.00
15-19 years	2004	0.01
15-19 years	2005	0.00
15-19 years	2006	0.00
15-19 years	2007	0.00
15-19 years	2008	0.01
15-19 years	2009	0.00
15-19 years	2010	0.00
15-19 years	2011	0.00
15-19 years	2012	0.00
15-19 years	2013	0.01
15-19 years	2014	0.00
15-19 years	2015	0.00
15-19 years	2016	0.00
15-19 years	2017	0.00
15-19 years	2018	0.00
15-19 years	2019	0.00
15-19 years	2020	0.00
15-19 years	2021	0.00
20-29 years	2000	0.01
20-29 years	2001	0.01
20-29 years	2002	0.01
20-29 years	2003	0.01
20-29 years	2004	0.00
20-29 years	2005	0.00
20-29 years	2006	0.00
20-29 years	2007	0.00
20-29 years	2008	0.01
20-29 years	2009	0.01
20-29 years	2010	0.01
20-29 years	2011	0.01
20-29 years	2012	0.00
20-29 years	2013	0.01
20-29 years	2014	0.00
20-29 years	2015	0.01
20-29 years	2016	0.01
20-29 years	2017	0.00
20-29 years	2018	0.01
20-29 years	2019	0.00
20-29 years	2020	0.00
20-29 years	2021	0.01
30-39 years	2000	0.01
30-39 years	2001	0.01
30-39 years	2002	0.00
30-39 years	2003	0.00
30-39 years	2004	0.00
30-39 years	2005	0.01
30-39 years	2006	0.01
30-39 years	2007	0.00
30-39 years	2008	0.01
30-39 years	2009	0.01
30-39 years	2010	0.00
30-39 years	2011	0.00
30-39 years	2012	0.01
30-39 years	2013	0.01
30-39 years	2014	0.01
30-39 years	2015	0.01
30-39 years	2016	0.01
30-39 years	2017	0.01
30-39 years	2018	0.01
30-39 years	2019	0.01
30-39 years	2020	0.01
30-39 years	2021	0.01
40-49 years	2000	0.01
40-49 years	2001	0.01
40-49 years	2002	0.01
40-49 years	2003	0.01
40-49 years	2004	0.00
40-49 years	2005	0.02
40-49 years	2006	0.01
40-49 years	2007	0.01
40-49 years	2008	0.01
40-49 years	2009	0.01
40-49 years	2010	0.01
40-49 years	2011	0.01
40-49 years	2012	0.01
40-49 years	2013	0.01
40-49 years	2014	0.01
40-49 years	2015	0.01
40-49 years	2016	0.01
40-49 years	2017	0.01
40-49 years	2018	0.01
40-49 years	2019	0.02
40-49 years	2020	0.02
40-49 years	2021	0.01
50-59 years	2000	0.01
50-59 years	2001	0.01
50-59 years	2002	0.01
50-59 years	2003	0.01
50-59 years	2004	0.01
50-59 years	2005	0.01
50-59 years	2006	0.00
50-59 years	2007	0.01
50-59 years	2008	0.01
50-59 years	2009	0.01
50-59 years	2010	0.01
50-59 years	2011	0.01
50-59 years	2012	0.01
50-59 years	2013	0.01
50-59 years	2014	0.01
50-59 years	2015	0.01
50-59 years	2016	0.01
50-59 years	2017	0.01
50-59 years	2018	0.01
50-59 years	2019	0.02
50-59 years	2020	0.01
50-59 years	2021	0.01
60-69 years	2000	0.00
60-69 years	2001	0.01
60-69 years	2002	0.01
60-69 years	2003	0.01
60-69 years	2004	0.00
60-69 years	2005	0.00
60-69 years	2006	0.01
60-69 years	2007	0.01
60-69 years	2008	0.01
60-69 years	2009	0.01
60-69 years	2010	0.01
60-69 years	2011	0.01
60-69 years	2012	0.01
60-69 years	2013	0.01
60-69 years	2014	0.01
60-69 years	2015	0.01
60-69 years	2016	0.01
60-69 years	2017	0.01
60-69 years	2018	0.01
60-69 years	2019	0.01
60-69 years	2020	0.02
60-69 years	2021	0.01
70-79 years	2000	0.01
70-79 years	2001	0.01
70-79 years	2002	0.01
70-79 years	2003	0.00
70-79 years	2004	0.01
70-79 years	2005	0.00
70-79 years	2006	0.01
70-79 years	2007	0.01
70-79 years	2008	0.00
70-79 years	2009	0.01
70-79 years	2010	0.00
70-79 years	2011	0.00
70-79 years	2012	0.01
70-79 years	2013	0.00
70-79 years	2014	0.01
70-79 years	2015	0.00
70-79 years	2016	0.00
70-79 years	2017	0.01
70-79 years	2018	0.01
70-79 years	2019	0.00
70-79 years	2020	0.00
70-79 years	2021	0.01
80 years and above	2000	0.00
80 years and above	2001	0.00
80 years and above	2002	0.00
80 years and above	2003	0.00
80 years and above	2004	0.00
80 years and above	2005	0.00
80 years and above	2006	0.00
80 years and above	2007	0.01
80 years and above	2008	0.00
80 years and above	2009	0.00
80 years and above	2010	0.01
80 years and above	2011	0.00
80 years and above	2012	0.00
80 years and above	2013	0.00
80 years and above	2014	0.00
80 years and above	2015	0.00
80 years and above	2016	0.00
80 years and above	2017	0.00
80 years and above	2018	0.00
80 years and above	2019	0.00
80 years and above	2020	0.00
80 years and above	2021	0.00

**Table 10:** AAMR trend joinpoint analysis of chronic rheumatic heart disease for the variable sex during the period 2000-2021.

Sex	Year	AAMR/100,000
Males and Females	2000	1.24
Males and Females	2001	1.14
Males and Females	2002	1.06
Males and Females	2003	1.14
Males and Females	2004	1.12
Males and Females	2005	1.08
Males and Females	2006	1.13
Males and Females	2007	1.08
Males and Females	2008	1.18
Males and Females	2009	1.12
Males and Females	2010	0.99
Males and Females	2011	0.97
Males and Females	2012	0.93
Males and Females	2013	0.83
Males and Females	2014	0.83
Males and Females	2015	0.81
Males and Females	2016	0.80
Males and Females	2017	0.75
Males and Females	2018	0.78
Males and Females	2019	0.74
Males and Females	2020	0.67
Males and Females	2021	0.75
Males	2000	1.06
Males	2001	0.91
Males	2002	0.86
Males	2003	0.90
Males	2004	0.90
Males	2005	0.89
Males	2006	0.93
Males	2007	0.89
Males	2008	0.95
Males	2009	0.88
Males	2010	0.79
Males	2011	0.82
Males	2012	0.75
Males	2013	0.67
Males	2014	0.67
Males	2015	0.64
Males	2016	0.67
Males	2017	0.61
Males	2018	0.67
Males	2019	0.62
Males	2020	0.55
Males	2021	0.65
Females	2000	1.39
Females	2001	1.34
Females	2002	1.23
Females	2003	1.34
Females	2004	1.31
Females	2005	1.24
Females	2006	1.30
Females	2007	1.23
Females	2008	1.38
Females	2009	1.32
Females	2010	1.17
Females	2011	1.11
Females	2012	1.09
Females	2013	0.97
Females	2014	0.97
Females	2015	0.95
Females	2016	0.92
Females	2017	0.88
Females	2018	0.88
Females	2019	0.85
Females	2020	0.78
Females	2021	0.83

**Table 11:** AAMR trend joinpoint analysis of chronic rheumatic heart disease for the variable age for males during the period 2000-2021.

Age range	Year	AAMR/100,000
0-4 years	2000	0.00
0-4 years	2001	0.01
0-4 years	2002	0.00
0-4 years	2003	0.01
0-4 years	2004	0.00
0-4 years	2005	0.00
0-4 years	2006	0.00
0-4 years	2007	0.00
0-4 years	2008	0.01
0-4 years	2009	0.01
0-4 years	2010	0.00
0-4 years	2011	0.00
0-4 years	2012	0.00
0-4 years	2013	0.00
0-4 years	2014	0.00
0-4 years	2015	0.00
0-4 years	2016	0.00
0-4 years	2017	0.00
0-4 years	2018	0.00
0-4 years	2019	0.00
0-4 years	2020	0.00
0-4 years	2021	0.00
5-9 years	2000	0.01
5-9 years	2001	0.01
5-9 years	2002	0.01
5-9 years	2003	0.01
5-9 years	2004	0.00
5-9 years	2005	0.00
5-9 years	2006	0.00
5-9 years	2007	0.01
5-9 years	2008	0.01
5-9 years	2009	0.00
5-9 years	2010	0.00
5-9 years	2011	0.01
5-9 years	2012	0.00
5-9 years	2013	0.00
5-9 years	2014	0.00
5-9 years	2015	0.01
5-9 years	2016	0.01
5-9 years	2017	0.01
5-9 years	2018	0.00
5-9 years	2019	0.00
5-9 years	2020	0.00
5-9 years	2021	0.00
10-14 years	2000	0.02
10-14 years	2001	0.03
10-14 years	2002	0.02
10-14 years	2003	0.02
10-14 years	2004	0.03
10-14 years	2005	0.02
10-14 years	2006	0.02
10-14 years	2007	0.01
10-14 years	2008	0.02
10-14 years	2009	0.01
10-14 years	2010	0.01
10-14 years	2011	0.01
10-14 years	2012	0.01
10-14 years	2013	0.01
10-14 years	2014	0.01
10-14 years	2015	0.01
10-14 years	2016	0.00
10-14 years	2017	0.01
10-14 years	2018	0.01
10-14 years	2019	0.01
10-14 years	2020	0.00
10-14 years	2021	0.00
15-19 years	2000	0.05
15- 19 years	2001	0.04
15-19 years	2002	0.03
15-19 years	2003	0.04
15-19 years	2004	0.04
15-19 years	2005	0.03
15-19 years	2006	0.02
15-19 years	2007	0.03
15-19 years	2008	0.03
15-19 years	2009	0.02
15-19 years	2010	0.02
15-19 years	2011	0.03
15-19 years	2012	0.02
15-19 years	2013	0.02
15-19 years	2014	0.01
15-19 years	2015	0.02
15-19 years	2016	0.02
15-19 years	2017	0.01
15-19 years	2018	0.02
15-19 years	2019	0.01
15-19 years	2020	0.01
15-19 years	2021	0.01
20-29 years	2000	0.09
20-29 years	2001	0.09
20-29 years	2002	0.07
20-29 years	2003	0.08
20-29 years	2004	0.07
20-29 years	2005	0.09
20-29 years	2006	0.06
20-29 years	2007	0.07
20-29 years	2008	0.06
20-29 years	2009	0.06
20-29 years	2010	0.05
20-29 years	2011	0.05
20-29 years	2012	0.04
20-29 years	2013	0.03
20-29 years	2014	0.04
20-29 years	2015	0.04
20-29 years	2016	0.03
20-29 years	2017	0.04
20-29 years	2018	0.03
20-29 years	2019	0.03
20-29 years	2020	0.03
20-29 years	2021	0.03
30-39 years	2000	0.09
30-39 years	2001	0.08
30-39 years	2002	0.08
30-39 years	2003	0.10
30-39 years	2004	0.09
30-39 years	2005	0.07
30-39 years	2006	0.09
30-39 years	2007	0.07
30-39 years	2008	0.08
30-39 years	2009	0.07
30-39 years	2010	0.07
30-39 years	2011	0.05
30-39 years	2012	0.06
30-39 years	2013	0.04
30-39 years	2014	0.04
30-39 years	2015	0.05
30-39 years	2016	0.04
30-39 years	2017	0.04
30-39 years	2018	0.05
30-39 years	2019	0.04
30-39 years	2020	0.04
30-39 years	2021	0.04
40-49 years	2000	0.14
40-49 years	2001	0.11
40-49 years	2002	0.10
40-49 years	2003	0.14
40-49 years	2004	0.13
40-49 years	2005	0.12
40-49 years	2006	0.13
40-49 years	2007	0.13
40-49 years	2008	0.11
40-49 years	2009	0.10
40-49 years	2010	0.08
40-49 years	2011	0.10
40-49 years	2012	0.09
40-49 years	2013	0.07
40-49 years	2014	0.08
40-49 years	2015	0.08
40-49 years	2016	0.09
40-49 years	2017	0.06
40-49 years	2018	0.09
40-49 years	2019	0.08
40-49 years	2020	0.06
40-49 years	2021	0.07
50-59 years	2000	0.18
50-59 years	2001	0.15
50-59 years	2002	0.17
50-59 years	2003	0.15
50-59 years	2004	0.15
50-59 years	2005	0.15
50-59 years	2006	0.18
50-59 years	2007	0.16
50-59 years	2008	0.14
50-59 years	2009	0.16
50-59 years	2010	0.14
50-59 years	2011	0.14
50-59 years	2012	0.12
50-59 years	2013	0.12
50-59 years	2014	0.14
50-59 years	2015	0.11
50-59 years	2016	0.11
50-59 years	2017	0.10
50-59 years	2018	0.10
50-59 years	2019	0.12
50-59 years	2020	0.08
50-59 years	2021	0.11
60-69 years	2000	0.25
60-69 years	2001	0.21
60-69 years	2002	0.21
60-69 years	2003	0.18
60-69 years	2004	0.16
60-69 years	2005	0.21
60-69 years	2006	0.21
60-69 years	2007	0.20
60-69 years	2008	0.23
60-69 years	2009	0.21
60-69 years	2010	0.18
60-69 years	2011	0.21
60-69 years	2012	0.18
60-69 years	2013	0.18
60-69 years	2014	0.14
60-69 years	2015	0.14
60-69 years	2016	0.18
60-69 years	2017	0.15
60-69 years	2018	0.16
60-69 years	2019	0.13
60-69 years	2020	0.13
60-69 years	2021	0.15
70-79 years	2000	0.15
70-79 years	2001	0.12
70-79 years	2002	0.11
70-79 years	2003	0.12
70-79 years	2004	0.14
70-79 years	2005	0.13
70-79 years	2006	0.16
70-79 years	2007	0.15
70-79 years	2008	0.19
70-79 years	2009	0.17
70-79 years	2010	0.17
70-79 years	2011	0.16
70-79 years	2012	0.16
70-79 years	2013	0.13
70-79 years	2014	0.13
70-79 years	2015	0.12
70-79 years	2016	0.12
70-79 years	2017	0.11
70-79 years	2018	0.13
70-79 years	2019	0.13
70-79 years	2020	0.12
70-79 years	2021	0.13
80 years and above	2000	0.09
80 years and above	2001	0.06
80 years and above	2002	0.06
80 years and above	2003	0.06
80 years and above	2004	0.07
80 years and above	2005	0.06
80 years and above	2006	0.05
80 years and above	2007	0.07
80 years and above	2008	0.07
80 years and above	2009	0.07
80 years and above	2010	0.06
80 years and above	2011	0.06
80 years and above	2012	0.07
80 years and above	2013	0.07
80 years and above	2014	0.08
80 years and above	2015	0.06
80 years and above	2016	0.06
80 years and above	2017	0.07
80 years and above	2018	0.07
80 years and above	2019	0.07
80 years and above	2020	0.06
80 years and above	2021	0.08

**Table 12:** AAMR trend joinpoint analysis of chronic rheumatic heart disease for the variable age for females during the period 2000-2021.

Age range	Year	AAMR/100,000
0-4 years	2000	0.00
0-4 years	2001	0.00
0-4 years	2002	0.00
0-4 years	2003	0.00
0-4 years	2004	0.00
0-4 years	2005	0.01
0-4 years	2006	0.00
0-4 years	2007	0.00
0-4 years	2008	0.00
0-4 years	2009	0.00
0-4 years	2010	0.01
0-4 years	2011	0.00
0-4 years	2012	0.00
0-4 years	2013	0.00
0-4 years	2014	0.00
0-4 years	2015	0.00
0-4 years	2016	0.00
0-4 years	2017	0.00
0-4 years	2018	0.00
0-4 years	2019	0.00
0-4 years	2020	0.01
0-4 years	2021	0.00
5-9 years	2000	0.01
5-9 years	2001	0.01
5-9 years	2002	0.01
5-9 years	2003	0.01
5-9 years	2004	0.01
5-9 years	2005	0.01
5-9 years	2006	0.01
5-9 years	2007	0.01
5-9 years	2008	0.01
5-9 years	2009	0.00
5-9 years	2010	0.01
5-9 years	2011	0.01
5-9 years	2012	0.01
5-9 years	2013	0.01
5-9 years	2014	0.00
5-9 years	2015	0.00
5-9 years	2016	0.00
5-9 years	2017	0.00
5-9 years	2018	0.00
5-9 years	2019	0.00
5-9 years	2020	0.00
5-9 years	2021	0.00
10-14 years	2000	0.02
10-14 years	2001	0.02
10-14 years	2002	0.02
10-14 years	2003	0.02
10-14 years	2004	0.01
10-14 years	2005	0.02
10-14 years	2006	0.02
10-14 years	2007	0.01
10-14 years	2008	0.02
10-14 years	2009	0.01
10-14 years	2010	0.01
10-14 years	2011	0.00
10-14 years	2012	0.01
10-14 years	2013	0.00
10-14 years	2014	0.01
10-14 years	2015	0.01
10-14 years	2016	0.01
10-14 years	2017	0.01
10-14 years	2018	0.02
10-14 years	2019	0.01
10-14 years	2020	0.00
10-14 years	2021	0.00
15-19 years	2000	0.03
15-19 years	2001	0.04
15-19 years	2002	0.03
15-19 years	2003	0.03
15-19 years	2004	0.03
15-19 years	2005	0.02
15-19 years	2006	0.02
15-19 years	2007	0.03
15-19 years	2008	0.03
15-19 years	2009	0.02
15-19 years	2010	0.02
15-19 years	2011	0.01
15-19 years	2012	0.01
15-19 years	2013	0.01
15-19 years	2014	0.01
15-19 years	2015	0.01
15-19 years	2016	0.01
15-19 years	2017	0.01
15-19 years	2018	0.01
15-19 years	2019	0.01
15-19 years	2020	0.01
15-19 years	2021	0.01
15-19 years	2000	0.09
15-19 years	2001	0.07
15-19 years	2002	0.08
15-19 years	2003	0.08
15-19 years	2004	0.08
15-19 years	2005	0.08
15-19 years	2006	0.06
15-19 years	2007	0.06
15-19 years	2008	0.06
20-29 years	2009	0.06
20-29 years	2010	0.05
20-29 years	2011	0.05
20-29 years	2012	0.06
20-29 years	2013	0.03
20-29 years	2014	0.04
20-29 years	2015	0.04
20-29 years	2016	0.04
20-29 years	2017	0.04
20-29 years	2018	0.02
20-29 years	2019	0.03
20-29 years	2020	0.03
20-29 years	2021	0.03
30-39 years	2000	0.13
30-39 years	2001	0.12
30-39 years	2002	0.12
30-39 years	2003	0.10
30-39 years	2004	0.13
30-39 years	2005	0.11
30-39 years	2006	0.13
30-39 years	2007	0.09
30-39 years	2008	0.10
30-39 years	2009	0.12
30-39 years	2010	0.09
30-39 years	2011	0.08
30-39 years	2012	0.09
30-39 years	2013	0.08
30-39 years	2014	0.08
30-39 years	2015	0.07
30-39 years	2016	0.07
30-39 years	2017	0.07
30-39 years	2018	0.07
30-39 years	2019	0.06
30-39 years	2020	0.06
30-39 years	2021	0.04
40-49 years	2000	0.22
40-49 years	2001	0.21
40-49 years	2002	0.19
40-49 years	2003	0.23
40-49 years	2004	0.20
40-49 years	2005	0.19
40-49 years	2006	0.22
40-49 years	2007	0.19
40-49 years	2008	0.24
40-49 years	2009	0.23
40-49 years	2010	0.17
40-49 years	2011	0.17
40-49 years	2012	0.16
40-49 years	2013	0.16
40-49 years	2014	0.17
40-49 years	2015	0.14
40-49 years	2016	0.15
40-49 years	2017	0.12
40-49 years	2018	0.13
40-49 years	2019	0.13
40-49 years	2020	0.11
40-49 years	2021	0.11
50-59 years	2000	0.28
50-59 years	2001	0.28
50-59 years	2002	0.23
50-59 years	2003	0.26
50-59 years	2004	0.27
50-59 years	2005	0.26
50-59 years	2006	0.22
50-59 years	2007	0.26
50-59 years	2008	0.29
50-59 years	2009	0.26
50-59 years	2010	0.26
50-59 years	2011	0.22
50-59 years	2012	0.24
50-59 years	2013	0.20
50-59 years	2014	0.18
50-59 years	2015	0.19
50-59 years	2016	0.19
50-59 years	2017	0.18
50-59 years	2018	0.17
50-59 years	2019	0.19
50-59 years	2020	0.17
50-59 years	2021	0.16
60-69 years	2000	0.28
60-69 years	2001	0.32
60-69 years	2002	0.29
60-69 years	2003	0.32
60-69 years	2004	0.32
60-69 years	2005	0.28
60-69 years	2006	0.32
60-69 years	2007	0.31
60-69 years	2008	0.32
60-69 years	2009	0.31
60-69 years	2010	0.28
60-69 years	2011	0.29
60-69 years	2012	0.27
60-69 years	2013	0.25
60-69 years	2014	0.26
60-69 years	2015	0.25
60-69 years	2016	0.22
60-69 years	2017	0.22
60-69 years	2018	0.23
60-69 years	2019	0.22
60-69 years	2020	0.21
60-69 years	2021	0.24
70-79 years	2000	0.21
70-79 years	2001	0.17
70-79 years	2002	0.17
70-79 years	2003	0.19
70-79 years	2004	0.18
70-79 years	2005	0.17
70-79 years	2006	0.19
70-79 years	2007	0.19
70-79 years	2008	0.22
70-79 years	2009	0.21
70-79 years	2010	0.18
70-79 years	2011	0.18
70-79 years	2012	0.16
70-79 years	2013	0.14
70-79 years	2014	0.14
70-79 years	2015	0.16
70-79 years	2016	0.14
70-79 years	2017	0.15
70-79 years	2018	0.14
70-79 years	2019	0.13
70-79 years	2020	0.11
70-79 years	2021	0.15
80 years and above	2000	0.12
80 years and above	2001	0.10
80 years and above	2002	0.09
80 years and above	2003	0.10
80 years and above	2004	0.08
80 years and above	2005	0.09
80 years and above	2006	0.09
80 years and above	2007	0.09
80 years and above	2008	0.09
80 years and above	2009	0.09
80 years and above	2010	0.09
80 years and above	2011	0.08
80 years and above	2012	0.09
80 years and above	2013	0.09
80 years and above	2014	0.07
80 years and above	2015	0.07
80 years and above	2016	0.07
80 years and above	2017	0.08
80 years and above	2018	0.07
80 years and above	2019	0.07
80 years and above	2020	0.07
80 years and above	2021	0.08
